# Recent Research on Role of p53 Family in Small-Cell Lung Cancer

**DOI:** 10.3390/cancers17071110

**Published:** 2025-03-26

**Authors:** Minho Jeong, Kee-Beom Kim

**Affiliations:** 1 School of Life Science and Biotechnology, College of Natural Sciences, Kyungpook National University, Daegu 41566, Republic of Korea; 2 BK21 FOUR KNU Creative BioResearch Group, School of Life Sciences, Kyungpook National University, Daegu 41566, Republic of Korea; 3 KNU-G LAMP Project Group, KNU-Institute of Basic Sciences, School of Life Sciences, College of Natural Sciences, Kyungpook National University, Daegu 41566, Republic of Korea

**Keywords:** SCLC, tumor suppressor gene, p53, p63, p73

## Abstract

Small-cell lung cancer (SCLC) is a highly aggressive malignancy characterized by rapid proliferation, early metastasis, and frequent recurrence, which contribute to a poor prognosis. SCLC is defined by the near-universal inactivation of key tumor suppressor genes, notably TP53 and RB1, which play central roles in its pathogenesis and resistance to therapy. The p53 family of proteins, including p53, p63, and p73, is essential to maintaining cellular homeostasis and tumor suppression. TP53 mutations are almost ubiquitous in SCLC, leading to dysregulated apoptosis and cell cycle control. Moreover, p73 shows potential as a compensatory mechanism for p53 loss, while p63 has a minimal role in this cancer type. In this review, we explore the molecular and functional interplay of the p53 family in SCLC, emphasizing its members’ distinct yet interconnected roles in tumor suppression, immune modulation, and therapy resistance. We highlight emerging therapeutic strategies targeting these pathways, including reactivating mutant p53, exploiting synthetic lethality, and addressing immune evasion mechanisms. Furthermore, this review underscores the urgent need for novel, isoform-specific interventions to enhance treatment efficacy and improve patient outcomes in this challenging disease.

## Simple Summary

Small-cell lung cancer (SCLC) is an aggressive cancer with a poor prognosis, largely due to the inactivation of tumor suppressor genes such as TP53. The p53 family, including p53, p63, and p73, plays a crucial role in tumor suppression, with p73 showing potential to compensate for p53 loss. However, mutant p53 contributes to therapy resistance and immune evasion, making treatment challenging. Emerging strategies, such as reactivating mutant p53 and targeting immune checkpoints, offer hope for improving patient outcomes. Further research is needed to develop effective, isoform-specific therapies for SCLC.

## 1. Introduction

Within the spectrum of lung cancers, small-cell lung cancer (SCLC) represents a particularly malignant type, contrasting with non-small-cell lung cancer (NSCLC) in its morphological and histological features. Indeed, SCLC is characterized by rapid proliferation, a strong tendency for metastasis, and frequent recurrence, making it significantly more aggressive than NSCLC [1]. Histopathological features, including small, round-to-fusiform cells with scant cytoplasm, finely granular nuclear chromatin, and frequent nuclear molding, alongside a high mitotic rate and extensive intra-tumoral necrosis, make it a highly aggressive cancer type. Furthermore, SCLC shows a distinct immunohistochemical profile, including positive staining for low-molecular-weight keratins, high levels of Ki67 as a hallmark of its aggressive proliferation, and variable expression of neuroendocrine markers such as chromogranin, synaptophysin, and CD56 [2]. SCLC is also known for its high malignancy, meaning that the cancer is highly aggressive, characterized by rapid growth and early metastatic tendency. This is further exacerbated by genetic instability, which contributes to the cancer’s rapid progression and a poor prognosis. These molecular characteristics are further stratified into four major subtypes, SCLC-A, SCLC-N, SCLC-P, and SCLC-I, based on the expression of key transcription factors such as ASCL1, NEUROD1, and POU2F3, which reflect distinct tumor behaviors and potential treatment responses [3].

SCLC represents approximately 15% of all lung cancer cases; moreover, SCLC patients commonly exhibit respiratory symptoms such as coughing, breathlessness, or hemoptysis. Diagnostic imaging frequently reveals a centrally located lung mass, often accompanied by considerable involvement of the thoracic lymph nodes. Thus, about two thirds of patients are already found to have distant metastases at the initial diagnosis, commonly involving the opposite lung, brain, liver, adrenal glands, and bones [4]. The frequent occurrence of metastasis at diagnosis significantly restricts treatment options, and the development of resistance to subsequent therapies contributes to the widespread recurrence of the disease [5]. The genetic landscape of SCLC also presents another layer of complexity, with extensive chromosomal alterations and a high mutational burden. This typically includes near-ubiquitous mutations in key tumor suppressor genes, namely, *TP53* and *RB1*, both of which contribute substantially to the aggressive behavior of the cancer and represent major obstacles in the development of effective targeted therapeutic strategies [4,6]. The prognosis for SCLC remains poor, with five-year survival rates as low as 3.5% and 10-year survival rates of only 1.8% [7]. Despite advances in medical treatments from 1997 to 2017, the survival rates of SCLC patients have not shown substantial improvement, underscoring a critical gap in the effectiveness of current therapies and emphasizing the urgent need for innovative treatment approaches [8]. Longitudinal studies have indicated that patients undergoing concurrent chemotherapy and radiation therapy may have overall survival rates of 52.4% at three years and 41.8% at five years. However, despite these advances, the five-year survival rate for patients receiving aggressive therapy remains around 40%, highlighting persistent challenges in improving long-term outcomes in SCLC [9].

In preventing the development and progression of cancers such as SCLC, it is essential to discuss the role of the p53 family [10]. Comprising p53, p63, and p73, the p53 family plays a crucial role in tumor suppression and maintaining cellular homeostasis. Among these proteins, TP53 mutations are found in nearly all cases of SCLC and contribute significantly to its aggressive nature [11]. In its normal state, the p53 tumor suppressor protein acts as a major barrier against cancer initiation and progression by functioning as a sequence-specific transcription factor that activates or represses gene transcription in response to cellular stress. However, mutations in the *TP53* gene, the most common genetic alterations in human cancers, can promote the loss of these crucial tumor suppressive functions, thereby allowing a malignancy to develop [12,13]. The development and progression of SCLC are further propelled by the loss of Rb, another tumor suppressor, which, along with p53 inactivation, enables cells to proliferate uncontrollably, evade apoptosis, and accumulate oncogenic mutations. Targeted therapies, such as PARP and Aurora kinase inhibitors, are being developed to address the downstream effects of these genetic aberrations [14]. Mutations in both p53 and p73 occur frequently in SCLC patients, contributing to the aggressive nature and complexity of this cancer [15]. In contrast, the role of p63, another member of the p53 family, is primarily associated with epithelial cell maintenance and differentiation. While direct mutations in p63 are less commonly observed in SCLC, p63 still plays a vital role in modulating the tumor microenvironment and maintaining cellular integrity. Additionally, the interactions of p63 with p53 and p73 may indirectly influence cancer progression and the therapeutic response, suggesting that all members of the p53 family contribute in different capacities to tumor suppression and cellular homeostasis [16]. These insights highlight the importance of the p53 family in cancer prevention and progression, especially in aggressive cancers such as SCLC. However, a deeper understanding of the roles of p53, p63, and p73 is crucial to advancing research and developing targeted therapies that could improve patient outcomes.

## 2. Structural and Functional Insights into the p53 Family Proteins

**Figure 1:**
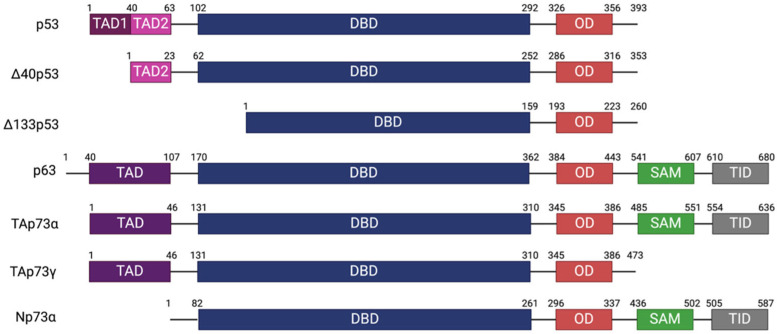
Domain structures of p53, p63, and p73 isoforms: The schematic illustrates the domain structures of p53 family isoforms, including p53, p63, and various p73 isoforms (TAp73α, TAp73γ, and Np73α). Each isoform contains distinct combinations of functional domains, influencing their role in tumor suppression and cellular regulation. The transactivation domain (TAD) facilitates transcriptional activation, while the DNA-binding domain (DBD) is essential to sequence-specific DNA interaction. The oligomerization domain (OD) supports tetramerization and structural stability. The SAM (sterile alpha motif) domain, found in p73α isoforms, mediates protein–protein interactions, whereas the TID (transcription inhibitory domain) plays a role in transcriptional repression. Differences in domain composition across isoforms highlight their functional divergence in cellular signaling and tumor biology.

The *p53*, *p63*, and *p73* genes have undergone multiple duplications throughout evolution, leading to the formation of a diverse family of proteins [17]. As a result, these three distinct genes, *p53*, *p63*, and *p73*, have been retained in most species, contributing to their versatile roles in various biological contexts [18,19]. The p53, p63, and p73 protein family consists of multiple domains, each with specific functions essential to maintaining cellular homeostasis and tumor suppression. These proteins notably include the transactivation domain (TAD), the DNA-binding domain (DNA BD), the oligomerization domain (OD), and the sterile alpha motif (SAM) domain (Figure 1) [20,21,22]. The DNA-binding domains of p53, p63, and p73 share remarkable structural similarity with conserved binding sequences across invertebrates and vertebrates. Specifically, human p53, p63, and p73 exhibit approximately 60–87% amino acid identity in their DNA-binding domains, with p63 and p73 sharing 87% identity, while p53 shares 55–63% identity with p63 and p73 [23]. This high degree of similarity underscores their functional overlap in transcriptional regulation. However, their C-terminal domains differ significantly, leading to distinct functional roles in transcriptional regulation and protein interactions [24]. Mutations in the DNA-binding domain (DNA BD) of p53 are especially significant in the context of SCLC. These are predominantly missense mutations, which impair the DNA-binding ability of p53 and compromise its tumor suppressor function. Additionally, such structural alterations hinder the interaction between p53 and DNA, disrupting the ability to induce cell cycle arrest and apoptosis [15]. In addition to mutations that hinder the tumor suppressor function of p53, the gene’s complexity is amplified by its generating diverse isoforms. In particular, the *p53* gene produces isoforms such as Δ40p53 and Δ133p53 through alternative splicing and post-translational modifications, contributing to functions such as DNA repair, replication, and cell survival regulation. These isoforms can modulate each other’s tumor suppressive activity, either enhancing or inhibiting it depending on the cellular context, and play crucial roles in determining cell fate under stress [25,26]. Mutant p53 (Onc-p53) in SCLC loses its tumor suppressor function and acquires new oncogenic properties that promote chemoresistance [27]. Similar to p53, p73 also exists in numerous isoforms, which adds to the complexity of tumor suppressive mechanisms and underscores the functional diversity within the p53 protein family. TP73 encodes more than 20 isoforms through alternative splicing and promoter usage, each contributing uniquely to both tumor suppressive and oncogenic functions [28]. The p73 protein exhibits multiple isoforms, including TAp73 and ΔNp73, generated through alternative promoter usage and splicing, each with distinct and sometimes opposing functions [29]. While TAp73 primarily acts as a tumor suppressor, ΔNp73 has been identified as an oncogene promoting cancer progression, metastasis, and therapeutic resistance. Recent studies on TP73 have demonstrated that alternative splicing leads to the isoform switch from TAp73α to TAp73γ, significantly impacting cancer progression. TAp73γ exhibits oncogenic properties, particularly by promoting cell proliferation and migration, while TAp73α maintains its tumor suppressive functions [30]. Adding to the structural complexity, both p63 and p73 contain a SAM domain, a feature notably absent in p53, which introduces an additional layer of regulatory intricacy. The SAM domain consists of five helices, four standard α-helices, and one 310 helix, which is thought to mediate specific protein–protein interactions [31,32,33]. Recent studies have demonstrated that p73 and p63 exhibit highly correlated expression patterns across various epithelial tissues, with co-localization in basal epithelial cell populations. This co-expression underscores the functional interplay between p73 and p63 in regulating cell differentiation, DNA repair, and tissue homeostasis [34]. In the case of p73, the C-terminal SAM domain has been shown to interact directly with the N-terminal region in MDM2, providing a crucial regulatory connection within the p53 family network. Notably, this interaction is predominantly facilitated by residues located in the α4 and α5 helices of SAMp73, with a substantial contribution from both α5 and the adjacent α4 helices. This binding feature of SAMp73 underscores its critical role in modulating p73 function, particularly in biological contexts where MDM2 plays a significant regulatory role [35]. Recent studies have shown that in the absence of wild-type p53, MDM2, MDMX, and p73 collaborate to regulate cell cycle progression. The depletion or inhibition of MDM2 or MDMX in p53-null cells results in cell cycle arrest, which is closely associated with reduced levels of E2F family activators and p73 [36]. Moreover, the p73α1 isoform, which lacks SAM domain-containing exon 12, has been found to uniquely modulate lipid metabolism and cancer cell survival. Specifically, p73α1 inhibits stearoyl-CoA desaturase-1 (SCD1), leading to increased saturated fatty acid levels, thereby reducing the viability of cancer cells [37]. Additionally, p73α1 significantly regulates Notch1 expression, a key mediator of inflammation, and increased Notch1 expression has been observed in inflamed mouse tissues [38].

## 3. Mechanism of Tumor Suppression and Immune Modulation by p53 Family

### 3.1. p53-Mediated Apoptosis and Cell Cycle Regulation

Notably, p53 is a key regulator that activates tumor suppressive pathways in response to various types of cellular stress, such as DNA damage, oxidative stress, and nutrient deprivation. Particularly, p53 exerts stringent control over the cell cycle and, when necessary, initiates apoptotic pathways to eliminate damaged cells, thereby mitigating cancer progression. This p53 tumor suppressive function is predominantly mediated by regulating downstream genes and proteins that govern critical cellular processes, such as DNA repair, apoptosis, and cell cycle arrest [39]. Under stress conditions, p53 induces autophagy, which removes damaged proteins and organelles within the cell, thereby playing a critical role in regulating the survival and death of cancer cells. This provides new insights into cancer progression and therapeutic strategies [40]. Further, p53 regulates vital transitions in the cell cycle, specifically, the G1/S and G2/M phases, thereby enforcing tumor suppressive actions through careful cell cycle control. In response to cellular damage, p53 induces cyclin-dependent kinase inhibitor p21 (Cip1/Waf1) expression, which subsequently inhibits the activity of cyclin–CDK complexes. This inhibition results in cell cycle arrest, allowing for sufficient time for DNA repair mechanisms to address the damage. When cellular damage is irreparable, p53 induces apoptosis to eliminate the damaged cell, thereby preventing potentially malignant cells from proliferating [41]. The *TP53* gene is frequently altered through chromosomal deletions, truncations, and missense mutations in SCLC, which occur in approximately 90% of tumors [15]. Loss of p53 function allows for the accumulation of genetic damage and unchecked proliferation, driving tumor progression and contributing to the aggressive nature of SCLC [14]. In addition to their role in tumor development, TP53 mutations are strongly associated with therapy resistance since p53 inactivation disrupts the apoptotic pathways required for chemotherapy and radiotherapy efficacy. This resistance, coupled with the genomic instability caused by p53 loss, exacerbates tumor heterogeneity and poor clinical outcomes in SCLC. Given its near-universal alteration in SCLC, TP53 is a key therapeutic target. Current strategies focus on reactivating mutant p53, leveraging synthetic lethal interactions, or enhancing the activity of related proteins, such as p63 and p73. These approaches hold promise for addressing the challenges of therapy resistance and improving outcomes for SCLC patients [15,42,43].

### 3.2. The General Roles of p73 and p63 in Tumor Suppression

Unlike p53, which is primarily recognized for its role in tumor suppression, p73 and p63 are mainly involved in developmental processes and epithelial differentiation, making their contributions to tumor suppression more complex and less direct. p63 and p73 knockout studies have revealed severe developmental abnormalities without a notable increase in cancer susceptibility, suggesting that their primary role lies in development rather than direct tumor suppression. However, certain p73 isoforms, such as TAp73, have been shown to compensate for p53 loss by inducing apoptosis in p53-deficient cancer cells, highlighting a context-dependent tumor suppressive role [44]. The *TP63* and *TP73* gene structures are complex, involving multiple promoters and splicing variations, which can produce both tumor suppressive and dominant-negative isoforms. This complexity contributes to their varied and sometimes contradictory roles in cancer biology [21,44,45]. For instance, the relative expression of TAp73 and ΔNp73 determines the balance between tumor suppression and promotion, with ΔNp73 often antagonizing the pro-apoptotic functions of TAp73 [45]. Moreover, their unique C-terminal extensions facilitate specialized protein–protein interactions with pathways critical to development, differentiation, and damage response, broadening their biological spectrum beyond classical tumor suppression [44]. Thus, p73 and p63 also involve diverse physiological processes, such as stem cell maintenance and neurogenesis, underscoring their multifunctionality. In addition, the ΔN isoforms of p63 and p73 can act as antagonists to p53, further complicating their roles in either promoting or suppressing tumor development [46].

### 3.3. Functional Synergy Within the p53 Family

The proteins of the p53 family (p53, p63, and p73) share structural similarities and interact functionally to form an integrated tumor suppression network. These interactions are often complementary, with each protein contributing to cellular integrity through mechanisms including apoptosis, cell cycle regulation, and differentiation [21]. For instance, p63 and p73 can compensate for the loss of p53 in certain cellular contexts, especially through apoptosis induction. However, these interactions can also be antagonistic. For example, the ΔN isoforms of p63 and p73 can inhibit the tumor suppressive functions of p53 and other family members. The balance between these isoforms and the formation of hetero-oligomeric complexes ultimately determines whether the result will be tumor suppression or progression [47,48]. Moreover, mutant p53 can interfere with the functions of p63 and p73 under certain conditions. Indeed, recent studies have shown that mutant p53 induces the amyloid-like aggregation of p63 and p73 within liquid droplets, leading to their sequestration and the loss of tumor suppressive function. This prion-like aggregation mechanism highlights the complex interplay within the p53 family in cancer biology, particularly how gain of function (GOF) mutations for mutant p53 can inhibit the tumor suppressive activities of p63 and p73 [49,50]. These interactions are key to understanding how the p53 family drives the unique pathology of SCLC. The loss of p53 and the dysregulation of p63 and p73 underlie the known aggressive phenotypes of SCLC, shaping its pathological characteristics and informing targeted therapies.

## 4. The Specific Roles of p53, p73, and p63 in SCLC Tumor Suppression

### 4.1. Inactivation of p53 and Its Consequences in SCLC

The loss of p53 impairs the ability of cells to manage DNA damage properly and leads to genomic instability, a hallmark of SCLC. This instability facilitates the accumulation of mutations that further enhance the aggressive phenotype of the tumor. Without functional p53, the typical pathways that eliminate or repair defective cells are compromised, resulting in cells that evade apoptosis and continue proliferating despite harboring substantial DNA abnormalities [51]. In particular, the high mutation rate of TP53 disrupts essential cellular mechanisms and impairs the ability of cells to repair DNA damage. This leads to increased genomic instability, which, in turn, accelerates the accumulation of further mutations, ultimately contributing to the aggressive progression of SCLC [52].

### 4.2. p73 as a Compensatory Mechanism for p53 Loss in SCLC

**Figure 2:**
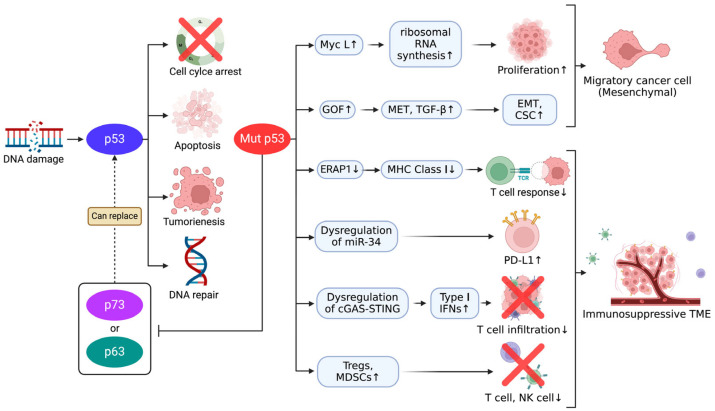
Mechanisms underlying mutant p53-induced tumor progression and immunosuppressive microenvironment. CSC (cancer stem cell), cGAS–STING (Cyclic GMP-AMP Synthase–Stimulator of Interferon Genes), EMT (epithelial-to-mesenchymal transition), ERAP (Endoplasmic Reticulum Aminopeptidase), GOF (gain of function), IFNs (interferons), MDSCs (Myeloid-Derived Suppressor Cells), MET (mesenchymal-to-epithelial transition), MHC class I (major histocompatibility complex class I), TGF-beta (Transforming Growth Factor-beta), and TME (tumor microenvironment).

In addition to p53, the other members of the p53 family, p63 and p73, also contribute to tumor suppression, although their roles are distinct. Indeed, p73, which shares significant structural similarity with p53, has been shown to induce apoptosis and contribute to cell cycle regulation, particularly in the absence of functional p53 [53]. Moreover, while not as frequently as those in p53, mutations in p73 have also been observed in SCLC, affecting its ability to compensate for the loss of p53 function [15]. Among the p73 isoforms, TAp73β, which retains the N-terminal transactivation domain, plays a role in this compensatory mechanism by activating genes involved in cell cycle arrest and apoptosis. Additionally, TAp73β has been shown to upregulate IL-1β, potentially contributing to the inflammatory microenvironment in SCLC [54], and to be able to oppose tumor angiogenesis by promoting HIF-1α degradation [55]. However, further research is needed to fully understand its specific contributions in SCLC. In SCLC, p73 contributes to tumor suppression, but its role varies depending on the isoform expressed. For instance, full-length p73α has been found to inhibit drug-induced apoptosis, indicating that p73 may support tumor suppression while potentially contributing to drug resistance, highlighting its complex role in SCLC [56]. Additionally, CDK4/6 inhibition has been shown to influence p73 activity independently of p53, as CDK4/6 inhibition suppresses p73 phosphorylation, enabling its translocation to the nucleus. Subsequently, p73 activates *DR5* transcription in the nucleus, a gene in the extrinsic apoptosis pathway, which can promote cell death even in p53-deficient cancer cells. This mechanism increases cancer cell sensitivity to immune checkpoint inhibitors and chemotherapy, suggesting that p73 can act as a compensatory mechanism in cancers with frequent p53 loss, such as SCLC [57]. Moreover, recent findings in NSCLC suggest that TAp73β enhances tumor suppression by promoting growth inhibition and increasing sensitivity to ferroptosis [58]. Furthermore, it has been suggested that the role of p73 in the cellular stress response, including regulating the DNA damage response via interactions with the ATM/ATR pathway, could enhance the apoptotic response in p53-deficient contexts. Indeed, the ambivalent role of p73, acting as both a tumor suppressor and, potentially, an oncogene, depends heavily on which isoform is expressed, highlighting its complexity in contributing to tumor behavior in SCLC [59,60]. The molecular mechanisms of mutant p53 in tumor progression and immune evasion are shown in Figure 2. These findings imply that p73 might be compensatory in SCLC, especially in the frequent absence of functional p53.

### 4.3. The Minimal Role of p63 in SCLC

In contrast, p63 appears to have a minimal role in SCLC, as it is generally not detected or only weakly expressed in this cancer type. A study of 23 SCLC cases showed that all were negative or rarely equivocal for p63 expression, whereas p63 was consistently expressed in poorly differentiated squamous cell carcinoma (PDSCC) [61]. Supporting this, in another study, it was reported that all tested SCLC samples were negative for p63 expression, highlighting that p63 is almost or entirely absent in SCLC [62]. In contrast, p63 is highly expressed in normal lung epithelial cells, particularly in bronchial reserve cells, where it plays a crucial role in maintaining the integrity of the epithelial tissue [63]. This lack of p63 expression in SCLC contrasts with its frequent expression in PDSCC, suggesting that p63 does not significantly contribute to tumor suppression in SCLC and emphasizing the different biological profiles between SCLC and other lung cancers where p63 plays a more prominent role [61,64].

## 5. The Role of the p53 Family in Immune Evasion and EMT in Cancer

### 5.1. Immune Evasion Mediated by p53 Family

Regulatory mechanisms governing immune checkpoint molecules such as PD-L1 and CTLA-4 involve intricate layers of control at the genetic, epigenetic, and post-translational levels [65]. Epigenetic changes, including DNA methylation and histone modification, play a crucial role in modulating PD-L1 expression in response to oncogenic signaling pathways [66]. Notably, in SCLC, PD-L1 expression has been reported in 26.0% of cases, highlighting its potential as a target for immune checkpoint inhibitor therapy [67]. Moreover, inflammatory cytokines such as IFN-γ upregulate PD-L1 by activating transcriptional pathways, further supporting immune escape. Post-translational modifications such as ubiquitination and glycosylation also influence the stability and localization of these molecules, enhancing their immunosuppressive effects within the tumor microenvironment [68]. Recent studies have demonstrated that mutant p53 plays a pivotal role in promoting immune evasion by suppressing the expression of major histocompatibility (MHC) class I molecules, thereby impairing antigen presentation. This mechanism is primarily driven by ERAP1 inhibition, which disrupts T-cell-mediated tumor surveillance. Furthermore, mutant p53 induces miR-34 dysregulation, increasing the expression of immune checkpoint molecules such as PD-L1, further reinforcing the immunosuppressive tumor microenvironment [69,70]. In parallel, p73 plays a complex dual role in cancer and immune regulation. While the TAp73 isoform promotes anti-tumor immunity by regulating immune-related genes and enhancing antigen presentation, the DNp73 isoform antagonizes these effects by promoting immune suppression and tumor progression. DNp73 has been linked to increased secretion of cytokines, including IL-6 and VEGF, which drive immune evasion by polarizing macrophages towards the immunosuppressive M2 phenotype and supporting angiogenesis. These dual roles of p73 add another layer of complexity to the immune evasion strategies utilized by the p53 family [71]. Adding to this complexity, the functional diversity of p73 isoforms is modulated by their interactions with the tumor microenvironment and other transcriptional regulators [72]. TAp73 enhances immune responses by promoting the resolution of inflammation and macrophage-mediated immunity, whereas DNp73 fosters an immunosuppressive tumor microenvironment. DNp73 also supports tumor progression by promoting epithelial-to-mesenchymal transition (EMT), cancer stemness, and angiogenesis, highlighting its oncogenic potential [72].

### 5.2. Mutant p53-Induced EMT and Its Implications for Therapeutic Resistance

The p53 family, notably, p53 and its homolog p73, is critical to suppressing tumor progression by maintaining cellular plasticity and preventing EMT. In addition, wild-type p53 contributes to immune modulation by regulating antigen presentation pathways. Wild-type p53 upregulates MHC class I expression by activating ERAP1 and TAP1, enhancing cytotoxic T-cell responses. Conversely, mutant p53 disrupts the cGAS–STING pathway, impairing type I interferon production and reducing CD8+ T-cell infiltration, facilitating immune evasion. Moreover, mutant p53 alters the tumor microenvironment by promoting the recruitment of immunosuppressive cells such as Tregs and MDSCs, further suppressing anti-tumor immunity [73]. Recent studies have demonstrated that the sequential loss of p53 and p73 induces stable EMT and enhances cancer stem cell (CSC) traits by activating the canonical Wnt signaling pathway, contributing to increased tumor aggressiveness and therapeutic resistance [74]. Specifically, p53 mutations promote increased mesenchymal-to-epithelial transition (MET) trafficking and signaling, enhancing cell motility and invasiveness, further escalating the aggressive behavior of cancer cells, and contributing to resistance against therapeutic interventions. In addition, mutant p53 proteins have been shown to promote a neomorphic gain of function (GOF), enhancing tumorigenic properties such as cell motility and invasiveness. This GOF activity is mediated through interactions with growth factor receptors, such as MET and TGF-β, further promoting EMT, cell motility, invasiveness, and CSC characteristics and contributing to an overall increase in tumor aggressiveness and therapeutic resistance [75,76].

These findings highlight the multifaceted roles of the p53 protein family in governing immune checkpoint regulation and immune escape mechanisms across various cancers. However, despite the near-ubiquitous presence of p53 mutations in SCLC, their specific impact on immune evasion, EMT, and therapeutic resistance in this aggressive subtype remains poorly understood. This gap in research limits the availability of SCLC-specific mechanistic insights, restricting the ability to cite focused studies on these topics and underscoring the urgent need for further investigation to fully comprehend and target these mechanisms in SCLC.

## 6. Therapeutic Strategies Targeting the p53 Family in SCLC

Therapeutic strategies targeting the p53 family in SCLC focus on restoring the tumor suppressive functions of p53 and addressing therapy resistance. Studies have shown that the biallelic loss of TP53 and RB1 is a hallmark of SCLC, with the common ancestral clone playing a central role in tumor progression and relapse [7]. Furthermore, co-alterations in TP73, CREBBP/EP300, and FMN2 have been found to exacerbate chemotherapy resistance, emphasizing the need for targeted approaches that address these genetic vulnerabilities [77]. The interplay between MYCL amplification and p53 inactivation is a critical driver in SCLC progression. MYCL promotes tumorigenesis by enhancing ribosomal biogenesis and protein synthesis, a particularly effective process when functional p53 is absent. Without the regulatory control of p53, MYCL-driven tumors exhibit unchecked growth and heightened dependency on ribosomal RNA synthesis, presenting a therapeutic vulnerability [78]. Experimental evidence demonstrates that RNA polymerase I inhibitors, such as CX-5461, selectively suppress MYCL-driven ribosomal RNA synthesis and induce autophagic cell death [79]. Meanwhile, CX-5461 has shown anti-tumor activity in clinical studies through both p53-dependent and p53-independent mechanisms, including activating DNA damage response pathways such as ATM/ATR. CX-5461 has demonstrated efficacy in mutant and wild-type TP53 malignancies, further validating its therapeutic potential. Preclinical models have shown significant tumor growth suppression following CX-5461 treatment, highlighting its potential as a therapeutic strategy for MYCL-amplified, p53-deficient SCLC tumors. CX-5461 exerts its effects independently of p53 status, making it particularly effective in SCLC tumors where p53 is inactivated [80]. Targeting MYCL-associated pathways such as ribosomal RNA synthesis offers a promising approach for combating MYCL-driven, p53-deficient SCLC tumors. However, the loss of p53 function creates vulnerabilities in ribosomal biogenesis and disrupts the ability of cells to maintain genomic stability and manage DNA damage. This genomic instability further enhances the aggressive phenotype of SCLC, emphasizing the interconnected roles of MYC-driven oncogenesis and p53 loss in shaping tumor behavior [79]. Therefore, exploring how the broader p53 family, including p73 and p63, compensates for these deficiencies may provide new insights into therapeutic strategies for SCLC.

Recent research has identified six significant pathways central to SCLC progression and treatment resistance: cell cycle and DNA repair, tumor development, cell metabolism, epigenetic regulation, tumor immunity, and angiogenesis. These pathways highlight the molecular vulnerabilities of SCLC and offer numerous therapeutic targets [81]. Among these, CHK1 inhibition has shown promise as a therapeutic approach. Moreover, CHK1 inhibitors, such as LY2606368 (prexasertib), have demonstrated significant activity in preclinical SCLC models, particularly when combined with agents such as cisplatin or PARP inhibitors, enhancing DNA damage and apoptosis. This strategy may help overcome therapy resistance, especially in platinum-refractory cases [82]. Recent studies have also highlighted the potential of combining BCL-2 inhibitors, such as venetoclax, with HSP90 inhibitors to target mutant p53 and overcome therapeutic resistance. This combination has shown promising results in preclinical models, inducing apoptosis in SCLC cells with high BCL-2 expression and mutant p53 [27]. Additionally, metabolic reprogramming and immunotherapy strategies, including PD-1/PD-L1 inhibitors, have shown the potential to improve outcomes when used in combination therapies [81]. The type of TP53 mutation, particularly those causing protein damage, has been associated with significantly shorter relapse-free survival, highlighting the critical impact of TP53 on therapeutic outcomes [77]. Mutant p53 (mutp53), prevalent in SCLC, not only loses its tumor suppressive functions but also gains oncogenic properties that drive cancer progression and metastasis. Recent studies have highlighted promising therapeutic strategies for targeting mutp53, including reactivating the wild-type function of p53 by using small molecules such as APR-246 and exploiting synthetic lethality through pathways including PARP inhibition. Moreover, vulnerabilities specific to mutp53, such as its interaction with ncRNAs and energy metabolism pathways, present novel opportunities for therapy. These approaches aim to counteract both the loss of function (LOF) and GOF characteristics of mutp53, offering a comprehensive framework for intervention [83]. Importantly, ReACp53, a peptide designed to inhibit mutant p53 aggregation, has shown promising results in reducing the aggressiveness of mutant p53 tumors by restoring the tumor suppressor activity of p53, reactivating mitochondrial apoptotic pathways, and enhancing the susceptibility of cancer cells to existing treatments. Recent advances, such as the development of ReACp53, a peptide targeting mutant p53 aggregation, have demonstrated potential in reversing the tumor-promoting effects of mutant p53 by restoring its suppressor functions, reactivating mitochondrial apoptotic pathways, and increasing the sensitivity of cancer cells to existing treatments. This suggests a potential therapeutic strategy for mitigating the negative impacts of mutant p53 on tumor progression and treatment resistance [70,84]. Since p53 is inactivated in over 90% of SCLC cases, the reactivation of p53 has shown potential in limiting tumor growth and metastasis by inducing senescence or cyclophilin-dependent necrosis. Emerging approaches include reactivating p53 with small molecules, such as APR-246, or targeting regulatory pathways involving MDM2 to stabilize p53. The interplay between p53 and other family members, such as p63 and p73, offers alternative pathways to enhance apoptosis and immune modulation. When combined with conventional therapies or immunotherapy, these strategies provide a promising framework for improving SCLC outcomes and overcoming therapeutic resistance [85,86,87,88]. Table 1 highlights the key therapeutic agents currently available for modulating the p53 pathway in SCLC, summarizing their mechanisms of action, targeted pathways, and clinical progress. These agents illustrate the breadth of strategies being explored to address the molecular vulnerabilities of SCLC and enhance therapeutic outcomes. Pharmacological enhancement of the tumor suppressive functions of p73 is considered a promising anticancer strategy, particularly in tumors with functional p53 deficiency or mutant p53 (mutp53). However, the complex biological characteristics associated with p73, including the presence of multiple isoforms with opposing functions, necessitate a cautious and nuanced approach to its therapeutic targeting [89]. For instance, while TAp73 overexpression has been observed in many solid tumors, negative regulatory interactions often inhibit its tumor suppressive potential. In SCLC, the major p73 isoforms, p73α and p73β, exhibit opposing biological roles, with p73α suppressing apoptosis and promoting cancer cell survival and therapy resistance, whereas p73β enhances apoptotic responses. The intricate interplay between these isoforms significantly influences therapeutic outcomes and poses a critical challenge to effectively utilizing p73 as a therapeutic target in SCLC. Consequently, research on directly targeting p73 in SCLC remains limited [56].

## 7. Conclusions

The critical role of the p53 family in SCLC progression, immune modulation, and therapy resistance underscores its potential as a key target for therapeutic intervention. In this review, we detailed the molecular mechanisms through which p53, p63, and p73 influence tumor suppression and their varied contributions to SCLC biology. The presented findings provide valuable insights into the aggressive nature of SCLC and open avenues for targeted treatments. The distinct yet interconnected roles of p53, p63, and p73 form a complex network of tumor suppressive mechanisms in SCLC. The near-universal loss of p53 function in SCLC leads to genomic instability and unchecked cellular proliferation. The compensatory mechanisms exhibited by p73, particularly its isoform TAp73, suggest the potential for targeted therapeutic strategies to mitigate the effects of p53 inactivation. In contrast, the minimal role of p63 in SCLC delineates its limited direct impact compared with its more prominent role in other cancer types.

Functional synergy and antagonism within the p53 family are critical to understanding SCLC pathology. Mutant p53 not only loses its tumor suppressive functions but also exerts dominant-negative effects by sequestering p63 and p73, further exacerbating tumor progression. These interactions emphasize the importance of addressing the GOF activities of mutant p53 in any therapeutic design. The dependency of SCLC on certain molecular pathways creates vulnerabilities that can be exploited therapeutically. For example, MYCL amplification coupled with p53 inactivation highlights a reliance on ribosomal RNA synthesis, making RNA polymerase I inhibitors, such as CX-5461, promising candidates. Similarly, targeting metabolic reprogramming through BCAT1 inhibition presents an avenue for disrupting the metabolic dependencies unique to SCLC. Emerging strategies targeting mutant p53 include small molecules such as APR-246 and peptides such as ReACp53, which aim to restore the tumor suppressive functions of p53 or neutralize its GOF effects. Combined with synthetic lethality strategies involving PARP and CHK1 inhibitors, these approaches are promising for addressing therapy resistance in SCLC.

The immune evasion mechanisms driven by mutant p53 and the dual roles of p73 isoforms present both challenges and opportunities. The suppression of antigen presentation and upregulation of immune checkpoint molecules by mutant p53 contributes to an immunosuppressive tumor microenvironment. Conversely, the potential of TAp73 to enhance immune responses contrasts with the immunosuppressive effects of ΔNp73, underscoring the need for isoform-specific therapeutic strategies. Immunotherapies targeting PD-L1 and CTLA-4, combined with agents modulating the functions of the p53 family, could enhance anti-tumor immune responses. These combinatorial approaches offer a promising avenue for overcoming immune evasion in SCLC.

The discussed findings emphasize the need for personalized therapeutic approaches that account for the molecular heterogeneity of SCLC. Meanwhile, future research should focus on developing therapies that selectively modulate p53 family isoforms to maximize tumor suppression and minimize off-target effects, exploring the synergistic potential of targeting p53 family pathways alongside immunotherapy and metabolic reprogramming and identifying biomarkers associated with early SCLC progression to enable timely detection and intervention. The multifaceted roles of the p53 family in SCLC progression, immune modulation, and therapy resistance highlight its significance as a therapeutic target. Thus, by leveraging insights into the molecular and functional diversity of p53, p63, and p73, novel strategies can be developed to address the aggressive nature of SCLC and improve patient outcomes. Therefore, the continued exploration of these pathways, in conjunction with translational studies, is pivotal to advancing SCLC treatment paradigms.

Small-cell lung cancer (SCLC) remains a highly aggressive malignancy, largely driven by the near-universal inactivation of TP53 and RB1. The p53 family, including p53, p63, and p73, plays a pivotal role in tumor suppression, with p73 emerging as a potential compensatory factor for p53 loss. However, the complexity of isoform-specific functions, particularly within p73, necessitates further investigation into targeted therapeutic strategies. The interplay between mutant p53 and immune evasion mechanisms underscores the challenges in overcoming therapy resistance, highlighting the need for innovative approaches such as synthetic lethality and immunotherapy-based interventions. Emerging therapeutic strategies targeting the p53 pathway, including reactivating mutant p53, inhibiting MYC-driven oncogenesis, and modulating immune responses, offer promising avenues for improving SCLC treatment outcomes. Despite significant advances, SCLC remains a difficult-to-treat disease, necessitating continued research into the isoform-specific targeting of the p53 family, combination therapies, and novel biomarkers for early detection. A deeper understanding of the molecular mechanisms governing p53 family interactions will be essential to developing more effective, personalized therapeutic strategies for SCLC patients.

## Figures and Tables

**Table 1:** p53-Related therapeutic drugs.

Name	Target	Mechanism of Action	References
Ganetespib	HSP90	Degrades mutant p53 and induces BIM expression	[46]
Venetoclax	BCL-2	Inhibits BCL-2 to induce apoptosis	[46,66,67]
Cisplatin	DNA crosslinking	Induces DNA damage, leading to apoptosis in cancer cells	[66,68]
Olaparib	PARP	Inhibits PARP, preventing DNA repair and causing cell death in p53-deficient tumors	[66,67,68]
Talazoparib		Strong PARP inhibition and DNA damage enhancement, causing cancer cell death	[67]
Prexasertib	CHK1	Inhibits CHK1, leading to increased DNA damage and apoptosis in p53-deficient cells	[67,68]
SRA-737		Targets CHK1 to enhance DNA damage effects in cancer cells	[67]
Nutlin-3a	MDM2	Inhibits the interaction between p53 and MDM2, reactivating wild-type p53	[69,70,71,72,73]
RG7112		Prevents p53 degradation by inhibiting MDM2	[69]
APR-246	Mutant p53	Restores the wild-type conformation of mutant p53 and induces apoptosis	[69,73]
COTI-2		Converts mutant p53 into its wild-type form and targets additional oncogenic pathways	[69,73]
Disulfiram		Promotes the degradation of wild-type and mutant p53 via the proteasome pathway	[69,73]
PRIMA-1		Restores the DNA-binding ability and wild-type conformation of mutant p53	[69]
HDAC inhibitors	Mutant p53/HDACs	Disrupts HDAC6/Hsp90/mutant p53 complexes and destabilizes mutant p53 proteins	[69,73]
